# ITS Sequencing Reveals the Changing Characteristics of Fungal Communities in Different Rice-Growing Substrates Under Salt Stress

**DOI:** 10.3390/biology14101456

**Published:** 2025-10-21

**Authors:** Hang Zhou, Xiaole Du, Yin Lin, Liming Zhao, Naijie Feng, Dianfeng Zheng

**Affiliations:** 1 College of Coastal Agricultural Sciences, Guangdong Ocean University, Zhanjiang 524000, China; 2 School of Tropical Agriculture and Forestry, Hainan University, Haikou 570100, China; 3 National Saline-Alkali Tolerant Rice Technology Innovation Center, Sanya 572019, China; 4 South China Center of National Saline-Alkali Tolerant Rice Technology Innovation Center, Zhanjiang 524000, China

**Keywords:** rice, soil, fungi, salt stress

## Abstract

**Simple Summary:**

Culture substrates with different physicochemical properties affect the initial structure of fungal communities and their response to salt stress. This study set up four culture substrates with different physicochemical properties, and the salt stress environment was artificially simulated. The changing characteristics of fungal communities in different rice-growing substrates under salt stress were revealed using ITS sequencing.

**Abstract:**

The impact of substrates with different physicochemical properties on the response of rhizosphere fungi in rice to salt stress has not been fully explored. The purpose of this study is to reveal the adaptation characteristics of fungal colonies to salt stress under different substrate conditions and the relationship between different properties of substrates and fungal colonies. Four different substrates were set by adjusting the sand, peat moss, and laterite ratio, with different bulk density, total porosity, and nutrient content. The same dose of sodium chloride solution was added to each substrate, and water was used as the control. The results showed that salt stress did not cause significant changes in the diversity and richness of fungal communities in different substrates. This study found that the responses of Ascomycota and *Penicillium* to salt stress varied depending on the substrate. The abundance of *Penicillium* was significantly positively correlated with total porosity (saline or non-saline conditions), but that of *Acrostalagmus* was significantly negatively correlated with total porosity under non-saline conditions. In addition, Lefse multi-level species difference discrimination analysis identified biomarkers in different treatments and revealed the core communities in response to substrate changes or salt stress. The results of this study contribute to a deeper understanding of the ecological functions of fungi.

## 1. Introduction

Soil microorganisms include many categories, such as bacteria, fungi, viruses, blue-green alga, etc. Some soil microorganisms are essential for recycling nutrients necessary for plant growth [1]. Fungi are an important part of microbial ecology. In nature, fungi participate in the decomposition of organic matter and provide nutrients for plant growth [2]. As biological agents that affect soil health, they play a very important role in plant protection [2,3].

At present, the total area of saline soil in the world is about 1.1 × 10^9^ hm^2^, and the degree of global soil salinization is still on the rise [4,5]. As one of the most serious factors affecting land degradation, salinity results in low soil microbial activity due to osmotic stress and toxic ions [6]. According to previous reports, soil respiration and fungal growth are inhibited under salt stress [7,8]. The presence of ionic and osmotic stress under salt stress leads to the accumulation of Na^+^ in cells and may damage membrane systems and cytosolic proteins [9]. However, in some cases, salt stress can positively affect fungi. For example, one study reported that the relative abundance of the fungal phylum Ascomycota increased under high salinity conditions [10]. In addition, some beneficial fungi play a role in improving plant salt tolerance. According to reports, *Penicillium* strains under salt stress produce gibberellins to promote plant growth [11,12]. *Aspergillus aculeatus* has been reported to enhance salt-stress tolerance in Perennial Ryegrass [13].

Substrates with different physicochemical properties may influence the initial structure of fungal communities and their response to salt stress. In this study, four rice culture substrates were set up by adjusting the ratios of river sand, peat moss, and laterite. ITS sequencing was used to reveal the response characteristics of fungal colonies in different substrates to salt stress. Studying how different substrates influence the response of fungal communities to salt stress can contribute to a deeper understanding of the ecological functions of fungi.

## 2. Materials and Methods

### 2.1. Test Materials

The variety used in this study was Xiangliangyou900, which was developed by Hunan Nianfeng Seed Technology Co., Ltd. (Changsha, China) and the Hunan Hybrid Rice Research Center. The rice cultivating device was a plastic bucket with an upper diameter × height × lower diameter of 28.5 × 23 × 19.3 cm. There were four types of substrates, namely, S1 (river sand); S2 (river sand: laterite: peat moss = 8:1:1); S3 (river sand: laterite: peat moss = 4:3:3); S4 (laterite: peat moss = 1:1). The setting of the substrate was based on the method of Zhou et al. with some modifications [14].

### 2.2. Experimental Design

This experiment was completed in the outdoor greenhouse of Guangdong Ocean University in 2024. The culture conditions were natural light, day/night temperatures of 31/25 ± 2 °C, and relative humidity of 60%. The sterilized seeds were soaked in fresh water for 24 h and then germinated in a wet state for 24 h. The germinated seeds were sown in seedling trays, cultured until they reached the 4-leaf stage, and then transplanted into substrate. In this study, each plastic bucket contained the same substrate volume (the substrate surface is 5 cm from the top of the bucket). The same volume of 0.3% NaCl aqueous solution (3.7 L) was added to each bucket once, and fresh water was used as a control. The water layer height of all treatments was maintained by adding fresh water during the subsequent cultivation process. All treatments were shown in Table 1.

This study used 0.63 g urea, 0.5625 g potassium chloride, and 0.9 g diammonium phosphate as base fertilizer (per bucket); 0.8975 g urea was used as tillering fertilizer (per bucket).

### 2.3. Substrate Parameters

Total phosphorus, total nitrogen, and total potassium were determined by the South Subtropical Crop Research Institute, China Academy of Tropical Agricultural Sciences. The measurement of these parameters referred to the method of Bao [15].

The bulk density was measured using a volumetric ring. The collected substrate samples were dried in an oven to a constant weight. The bulk density was measured using the following formula [16]:

Bulk density (BD) = dry weight/volume before drying

The total porosity was obtained by the following formula [17]:

Total porosity = (1 − bulk density/specific gravity) × 100%

Among them, the specific gravity is 2.65.

### 2.4. ITS Sequencing

#### 2.4.1. Sample Collection

Samples were collected between 5 and 10 cm below the substrate surface at the end of the jointing stage for physicochemical parameter determination. The samples from the rhizosphere were collected for ITS sequencing. Each treatment included three replicates (from three buckets).

#### 2.4.2. PCR Amplification and Sequencing Library Construction

The primer design for this study was shown in Table 2.

The PCR reaction system in this study included 10 × Buffer (2 μL), 2.5 mM dNTPs (2 μL), 5 μM forward primer (0.8 μL), 5 μM reverse primer (0.8 μL), rTaq polymerase (0.2 μL), BSA (0.2 μL), and template DNA (10 ng). ddH_2_O was added to make the system reach 20 μL.

Amplification procedure: Pre-denaturation at 95 °C for 3 min; 35 cycles (30 s at 95 °C, 30 s at 55 °C, 45 s at 72 °C). Stable extension at 72 °C for 10 min, and storage at 4 °C. PCR instrument was ABI GeneAmp^®^ 9700 (Applied Biosystems, Waltham, MA, USA).

In addition, the NEXTFLEX Rapid DNA-Seq kit (Bioo Scientific, Austin, TX, USA) was used to construct the library of the purified PCR products. This study used the Illumina PE300 platform (Illumina, San Diego, CA, USA) for sequencing.

In this study, alpha diversity index was used to analyze the richness, diversity, and evenness of microbial community; the difference test method was the Kruskal–Wallis rank sum test. In addition, the species composition of different treatments at each classification level (such as phylum and genus) was obtained based on the results of taxonomic analysis; the R language (version 3.3.1) tool was used to draw pictures.

All the above work was completed by Shanghai Meiji Biopharmaceutical Technology Co., Ltd. (Shanghai, China).

### 2.5. Statistical Analysis

This study used Origin 2021 to produce figures. SPSS 27 was used for one-way analysis of variance. This study used EndNote 20 to insert literature and Open Grammarly (v1.2.198.1762) to correct English grammar.

## 3. Results

### 3.1. Bulk Density, Total Porosity, and Nutrient Content of Different Substrates

**Figure 1:**
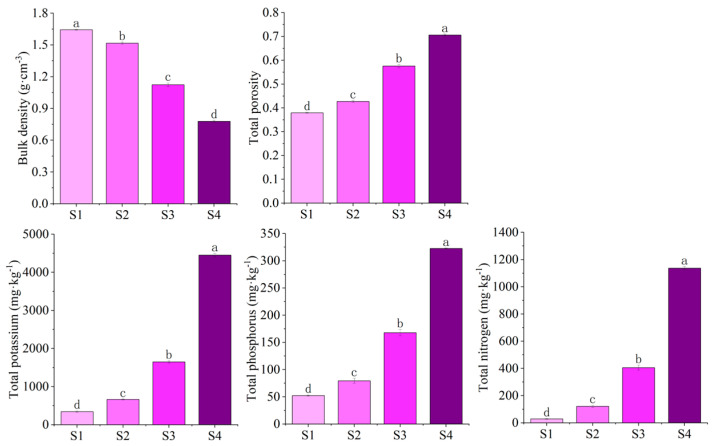
Bulk density, total porosity, and nutrient content of different substrates. The value corresponding to each column is the mean ± standard error (n = 3; *p* < 0.05). Different letters represent significant differences.

In this study, the bulk density of S1 was the highest, followed by S2, S3, and S4; there were significant differences among the different treatments. In contrast, the total porosity showed an increasing trend, i.e., S1 < S2 < S3 < S4; there were significant differences among different treatments. The total nitrogen, total phosphorus, and total potassium content showed S1 < S2 < S3 < S4, with significant differences among different treatments. As the proportion of peat moss and laterite increased, the bulk density gradually decreased, and the total porosity and nutrient content increased, indicating significant differences in the physical structure and fertility of different substrates (Figure 1).

### 3.2. Dilution Curve Analysis

**Figure 2:**
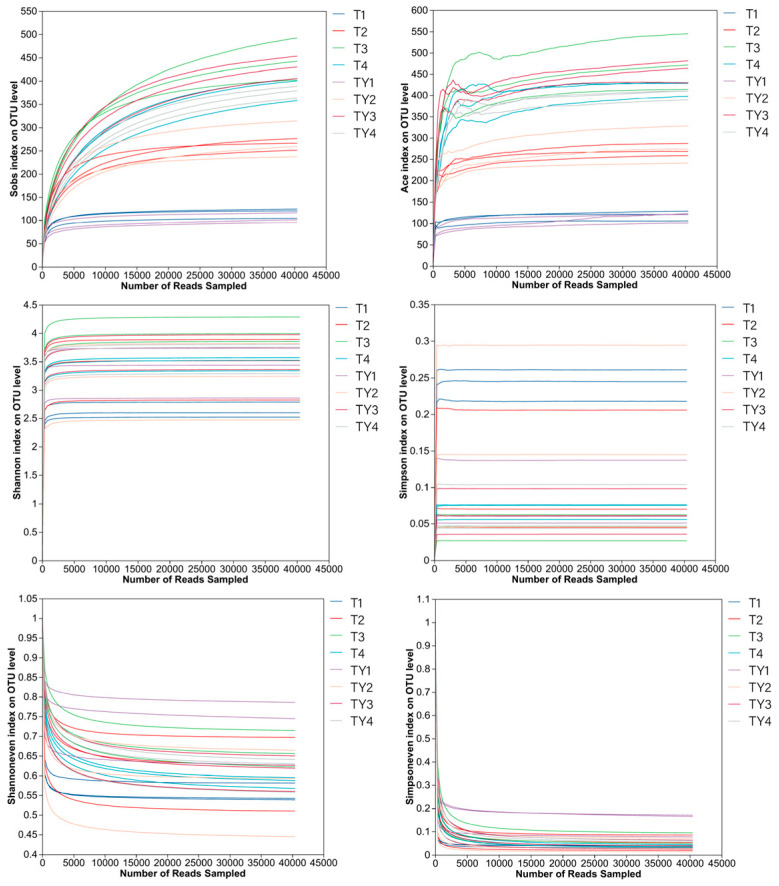
Dilution curve analysis. The horizontal axis represents the amount of sequencing data; the vertical axis represents the number of species observed or the diversity index.

The results of this study showed that the dilution curve was approaching a flat state, indicating that the detection ratio of the microbial community in the substrate sample was close to saturation, and the current sequencing volume could cover most of the species in the sample. The current quantity of sequencing data was sufficient to reflect the fungal community composition characteristics of the sample. This result was suitable for subsequent community comparison and functional analysis (Figure 2).

### 3.3. Alpha Diversity Analysis

#### 3.3.1. Alpha Diversity Analysis of Different Comparison Groups

**Figure 3:**
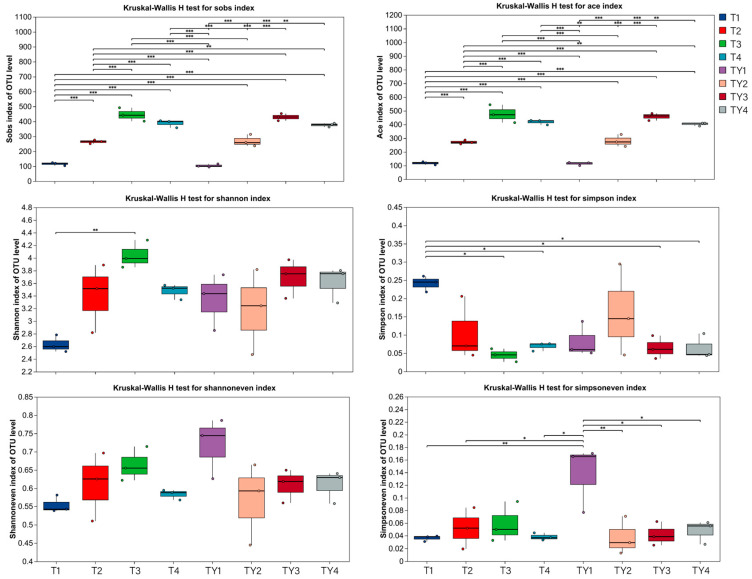
Index differences between different groups. The horizontal axis is the treatment name, and the vertical axis is the index range. * 0.01 < *p* ≤ 0.05, ** 0.001 < *p* ≤ 0.01, *** *p* ≤ 0.001.

This study showed no significant differences in Sobs, Ace, Simpson, and Shannon indices between T1 and TY1, T2 and TY2, T3 and TY3, and T4 and TY4. In addition, the Simpsoneven index of TY1 was significantly higher than that of T1, indicating that the community uniformity of TY1 was higher than that of T1 (Figure 3).

#### 3.3.2. Alpha Diversity Analysis of Different Non-Salt Treatments

As shown in Figure 3, the Sobs and Ace indices of T1 were significantly lower than those of T2, T3, and T4; T2 was significantly lower than T3 and T4. The Shannon index of T1 was significantly lower than that of T3, and the Simpson index of T1 was significantly higher than those of T3 and T4. There were no significant differences in the Shannoneven and Simpsoneven indices among the four treatments T1, T2, T3, and T4.

#### 3.3.3. Alpha Diversity Analysis of Different Salt Treatments

The Sobs and Ace indices of TY1 were significantly lower than those of TY2, TY3, and TY4. The Sobs and Ace indices of TY2 were significantly lower than those of TY3 and TY4. There were no significant differences in the Shannon and Simpson indices among TY1, TY2, TY3, and TY4. In addition, the Simpsoneven index of TY1 was significantly higher than that of TY2, TY3, and TY4 (Figure 3).

### 3.4. Species Composition Analysis

#### 3.4.1. Analysis of Community Composition of Different Comparison Groups

In the TY1/T1 comparison group, the relative abundance of Ascomycota increased, and that of unclassified_k__Fungi decreased. In the TY2/T2 comparison group, the relative abundance of Ascomycota increased slightly, and that of unclassified_k__Fungi decreased slightly. In the TY3/T3 comparison group, the relative abundances of Ascomycota and unclassified_k__Fungi decreased, and that of Basidiomycota increased. In the TY4/T4 comparison group, the relative abundance of Ascomycota increased, and that of Basidiomycota decreased.

**Figure 4:**
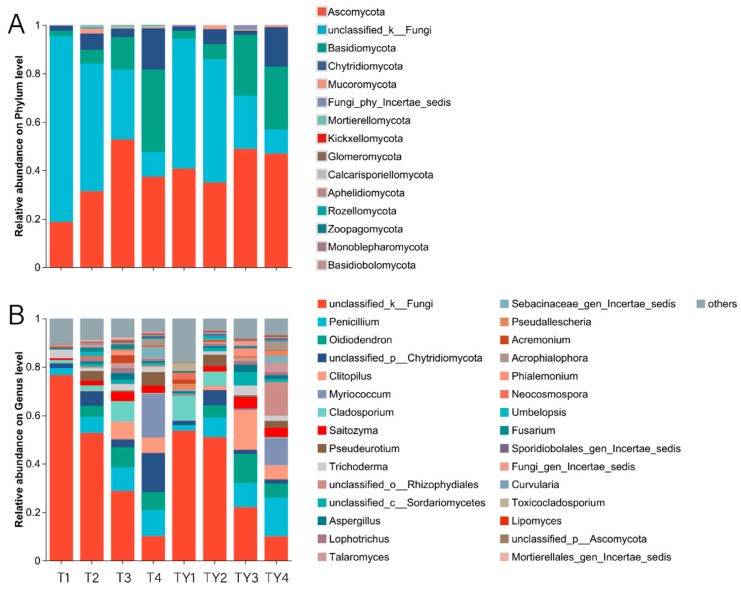
Community composition analysis bar chart. The horizontal axis is the treatment name, and the vertical axis is the proportion of the species in the sample. (**A**) at the phylum level. (**B**) at the genus level.

At the genus level, the relative abundance of unclassified_k__Fungi decreased, and that of *Cladosporium* increased in the TY1/T1 comparison group. In the TY2/T2 comparison group, the relative abundance of unclassified_k__Fungi decreased slightly, and that of *Penicillium* increased slightly; the relative abundance of *Cladosporium* increased. In the TY3/T3 comparison group, the relative abundances of unclassified_k__Fungi and *Cladosporium* decreased, and those of *Oidiodendron*, *Clitopilus*, and unclassified_c__*Sordariomycetes* increased. In the TY4/T4 comparison group, the relative abundances of *Penicillium* and unclassified_o__*Rhizophydiales* increased, and those of *Oidiodendron*, unclassified_p__*Chytridiomycota*, and *Myriococcum* decreased (Figure 4).

#### 3.4.2. Analysis of Community Composition of Different Non-Salt Treatments

At the phylum level, the dominant microbial communities in T1 and T2 were Ascomycota and unclassified_k__Fungi, and those in T3 were Ascomycota, unclassified_k__Fungi, and Basidiomycota. In T4, the dominant microbial communities were Ascomycota, unclassified_k__Fungi, Basidiomycota, and Chytridiomycota. The relative abundance of Ascomycota was the highest in T3, followed by that in T4. The relative abundance of unclassified_k__Fungi showed a trend of T1 > T2 > T3 > T4. The relative abundance of Basidiomycota showed a trend of T1 < T2 < T3 < T4. The relative abundance of Chytridiomycota in T4 was higher than that in the other three treatments.

At the genus level, the dominant microbial community of T1 was unclassified_k__Fungi. The dominant microbial communities of T2 were unclassified_k__Fungi, *Penicillium*, *Oidiodendron*, unclassified_p__*Chytridiomycota*, and *Pseudeurotium*. The dominant microbial communities of T3 were unclassified_k__Fungi, *Penicillium*, *Oidiodendron*, *Clitopilus*, and *Cladosporium*. The dominant microbial communities of T4 were unclassified_k__Fungi, *Penicillium*, *Oidiodendron*, unclassified_p__*Chytridiomycota*, *Clitopilus*, and *Myriococcum*. The relative abundance of *Penicillium* showed a trend of T1 < T2 < T3 < T4. The relative abundances of *Oidiodendron*, *Cladosporium*, and *Clitopilus* in T3 were higher than those in the other three treatments (Figure 4).

#### 3.4.3. Analysis of Community Composition of Different Salt Treatments

The dominant microbial communities in TY1 and TY2 were Ascomycota and unclassified_k__Fungi, and those in TY3 were Ascomycota, unclassified_k__Fungi, and Basidiomycota. The dominant microbial communities in TY4 were Ascomycota, unclassified_k__Fungi, Basidiomycota, and Chytridiomycota. Compared with other treatments, Ascomycota had the highest relative abundance in TY3. The relative abundance of unclassified_k__Fungi showed a decreasing trend, i.e., TY1 > TY2 > TY3 > TY4. The relative abundance of Basidiomycota showed a changing pattern of TY1 < TY2 < TY3 < TY4. The relative abundance of Chytridiomycota in TY4 was higher than that in the other three treatments.

At the genus level, the dominant microbial communities in TY1 were unclassified_k__Fungi and *Cladosporium*. In TY2, they were unclassified_k__Fungi, *Penicillium*, *Oidiodendron*, unclassified_p__*Chytridiomycota*, and *Cladosporium*. In TY3, the dominant microbial communities were unclassified_k__Fungi, *Penicillium*, *Oidiodendron*, and *Clitopilus*. In TY4, they were unclassified_k__Fungi, *Penicillium*, *Oidiodendron*, *Clitopilus*, *Myriococcum*, and unclassified_o__*Rhizophydiales*. The relative abundance of unclassified_k__Fungi followed the rule of TY1>TY2>TY3>TY4; the relative abundance of *Penicillium* was just the opposite. Compared with other treatments, *Cladosporium* had the highest relative abundance in TY1. The relative abundances of *Oidiodendron* and *Clitopilus* in TY3 were higher than those in the other three treatments (Figure 4).

### 3.5. Beta Diversity Analysis

**Figure 5:**
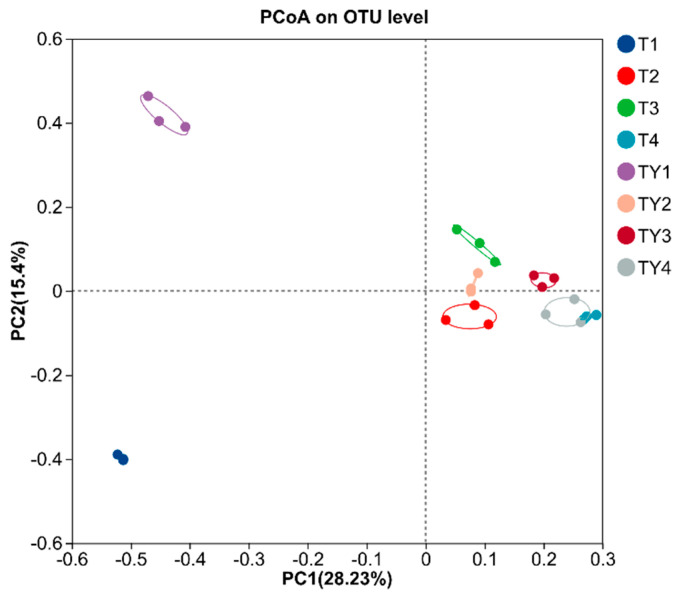
PCoA. The percentage represents the explanation value of the principal coordinate axis for the sample composition difference. The closer the two sample points are, the more similar the species composition of the two samples is.

PCoA showed that different treatments were separated, and the three samples of each treatment clustered together, indicating that the community composition differed between the different treatments and the experiment had good repeatability (Figure 5).

### 3.6. Lefse Multi-Level Species Difference Discriminant Analysis

**Figure 6:**
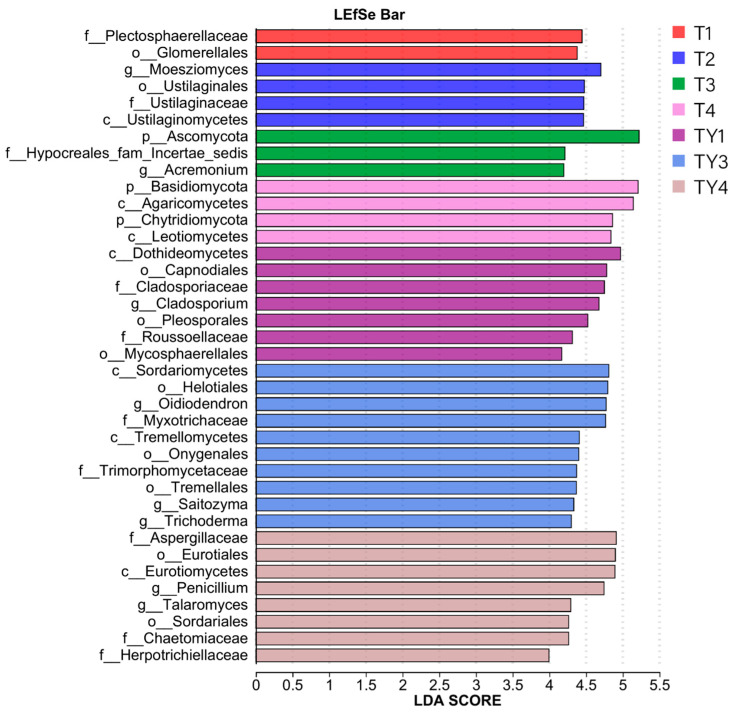
LDA discriminant bar chart. The larger the LDA score, the greater the influence of species abundance on the difference effect.

As shown in Figure 6 (LDA threshold > 2), o__Glomerellales and f__Plectosphaerellaceae were significantly enriched in T1. c__Ustilaginomycetes, f__Ustilaginaceae, o__Ustilaginales, and g__*Moesziomyces* were significantly enriched in T2. g__*Acremonium*, f__Hypocreales_fam_Incertae_sedis, and p__Ascomycota were significantly enriched in T3. In T4, c__Leotiomycetes, p__Chytridiomycota, c__Agaricomycetes, and p__Basidiomycota were significantly enriched. In TY1, o__Mycosphaerellales, f__Roussoellaceae, o__Pleosporales, g__*Cladosporium*, f__Cladosporiaceae, o__Capnodiales, and c__Dothideomycetes were significantly enriched. g__*Trichoderma*, g__*Saitozyma*, o__Tremellales, f__Trimorphomycetaceae, o__Onygenales, c__Tremellomycetes, f__Myxotrichaceae, g__*Oidiodendron*, o__Helotiales, and c__Sordariomycetes were significantly enriched in TY3. f__Herpotrichiellaceae, f__Chaetomiaceae, o__Sordariales, g__*Talaromyces*, g__*Penicillium*, c__Eurotiomycetes, o__Eurotiales, and f__Aspergillaceae were significantly enriched in TY4.

### 3.7. FUNGuild Function Prediction

#### 3.7.1. FUNGuild Function Prediction of Different Comparison Groups

**Figure 7:**
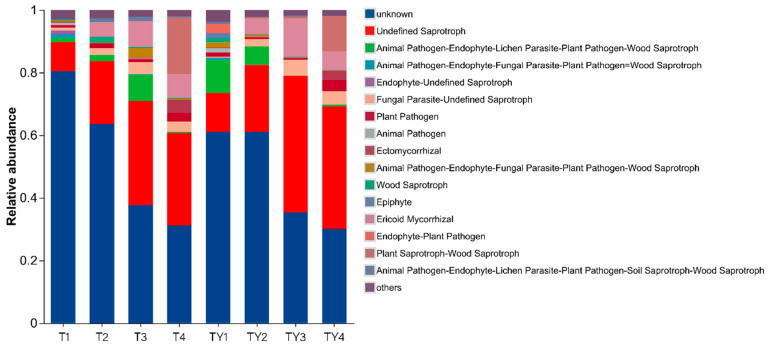
FUNGuild functional classification statistical bar chart. The vertical axis is the abundance ratio of Guild in different treatments, and the horizontal axis represents different treatments.

Compared with T1, the relative abundances of Endophyte–Plant Pathogen, Animal Pathogen–Endophyte–Lichen Parasite–Plant Pathogen–Wood Saprotroph, and Undefined Saprotroph increased in TY1. The relative abundances of Ericoid Mycorrhizal and Animal Pathogen–Endophyte–Lichen Parasite–Plant Pathogen–Wood Saprotroph increased in TY2 compared with T2. The relative abundances of Ericoid Mycorrhizal and Undefined Saprotroph in TY3 were higher than those in T3, while the relative abundances of Animal Pathogen–Endophyte–Fungal Parasite–Plant Pathogen–Wood Saprotroph and Animal Pathogen-Endophyte–Lichen Parasite–Plant Pathogen–Wood Saprotroph were lower than those in T3. Compared with T4, the relative abundance of Plant Saprotroph–Wood Saprotroph decreased in TY4 (Figure 7).

#### 3.7.2. FUNGuild Function Prediction of Different Non-Salt Treatments

The relative abundance of Plant Saprotroph–Wood Saprotroph in T4 was much greater than that in T1, T2, and T3. The relative abundance of Ericoid Mycorrhizal in T2, T3, and T4 was much greater than that in T1. The relative abundance of Ectomycorrhizal in T4 was higher than that in T1, T2, and T3. The relative abundance of Plant Pathogen was the highest in T4, followed by that in T2. The relative abundance of Animal Pathogen–Endophyte–Lichen Parasite–Plant Pathogen–Wood Saprotroph in T3 was higher than that in T1, T2, and T4. Compared with other treatments, the relative abundance of Undefined Saprotroph in T3 was the highest (Figure 7).

#### 3.7.3. FUNGuild Function Prediction of Different Salt Treatments

The relative abundances of Plant Saprotroph–Wood Saprotroph, Ectomycorrhizal, and Plant Pathogen in TY4 were much higher than those in TY1, TY2, and TY3. The relative abundances of Endophyte–Plant Pathogen, Epiphyte, Wood Saprotroph, Animal Pathogen–Endophyte–Fungal Parasite–Plant Pathogen–Wood Saprotroph, and Animal Pathogen–Endophyte–Lichen Parasite–Plant Pathogen–Wood Saprotroph in TY1 were higher than those in TY2, TY3, and TY4. In addition, the relative abundances of Undefined Saprotroph and Ericoid Mycorrhizal showed TY3 > TY4 > TY2 > TY1 (Figure 7).

### 3.8. Combined Analysis of Fungal Colonies and Environmental Factors

#### 3.8.1. Canonical Correspondence Analysis (CCA)

**Figure 8:**
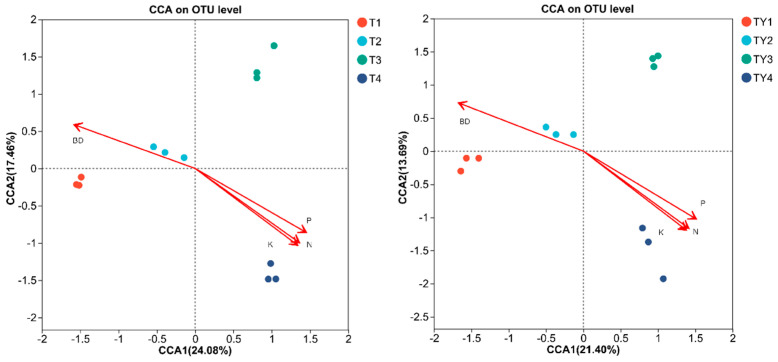
CCA Plot. The length of the environmental factor arrows represents the degree of influence (explanatory power) of the environmental factor on the species data. The angle between the environmental factor arrows represents positive or negative correlation (acute angle: positive correlation; obtuse angle: negative correlation; right angle: no correlation). The sample point is projected onto the arrows of the quantitative environmental factor, and the distance from the projection point to the origin represents the relative impact of the environmental factor on the distribution of the sample community.

As shown in Figure 8 below, under non-salt conditions, bulk density had the least relative impact on the microbial community distribution of T2 compared to other physicochemical parameters. The physicochemical properties had the greatest relative impact on the microbial community distribution of T3 compared to other treatments. The results were the same under salt stress.

#### 3.8.2. Correlation Analysis

**Figure 9:**
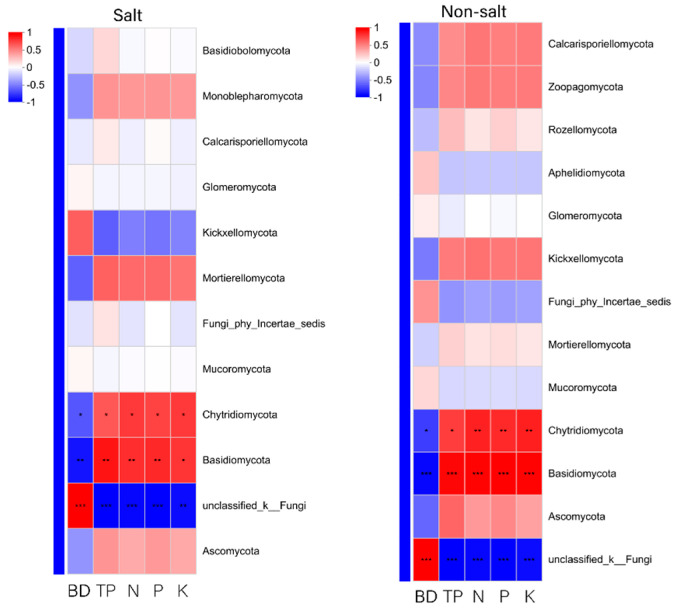
Heat map of correlation between fungal colonies and environmental factors at the phylum level. * 0.01 < *p* ≤ 0.05, ** 0.001 < *p* ≤ 0.01, *** *p* ≤ 0.001.

At the phylum level, Chytridiomycota and Basidiomycota were significantly positively correlated with total porosity, total nitrogen, total phosphorus, and total potassium, and significantly negatively correlated with bulk density under saline or non-saline conditions (Figure 9).

At the genus level, *Penicillium*, *Oidiodendron*, *Myriococcum*, *Acrophialophora*, and *Solicoccozyma* were significantly positively correlated with total porosity, total nitrogen, total phosphorus, and total potassium, and significantly negatively correlated with bulk density under non-salt conditions. *Condenascus* was significantly positively correlated with total porosity, total nitrogen, and total phosphorus, and significantly negatively correlated with bulk density. *Acrostalagmus* was significantly negatively correlated with total porosity and significantly positively correlated with bulk density. *Lipomyces* were significantly positively correlated with total porosity and total phosphorus and significantly negatively correlated with bulk density. *Saitozyma* was significantly positively correlated with total porosity and significantly negatively correlated with bulk density.

**Figure 10:**
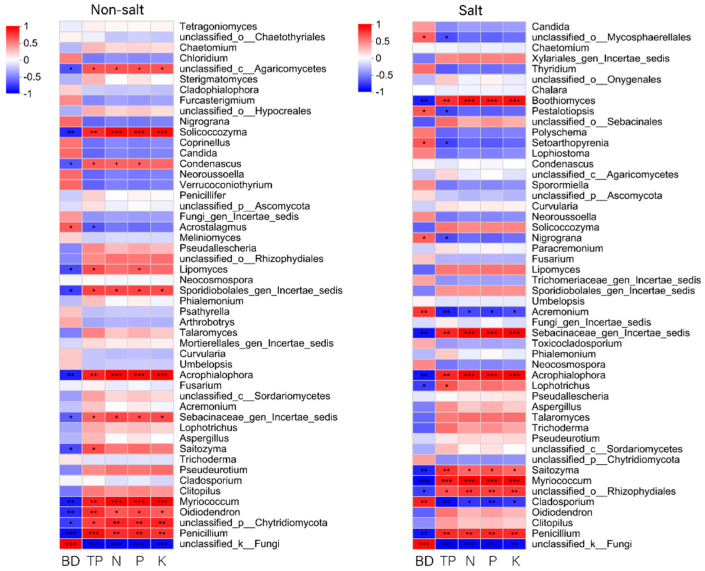
Heat map of the correlation between fungal colonies and environmental factors at the genus level. * 0.01 < *p* ≤ 0.05, ** 0.001 < *p* ≤ 0.01, *** *p* ≤ 0.001.

Under salt stress, *Penicillium*, *Myriococcum*, *Saitozyma*, *Acrophialophora*, and *Boothiomyces* were significantly positively correlated with total porosity, total nitrogen, total phosphorus, and total potassium, and significantly negatively correlated with bulk density. *Pestalotiopsis*, *Setoarthopyrenia*, and *Nigrograna* were significantly negatively correlated with total porosity and significantly positively correlated with bulk density. *Lophotrichus* was significantly positively correlated with total porosity and significantly negatively correlated with bulk density. *Acremonium* and *Cladosporium* were significantly negatively correlated with total porosity, total nitrogen, total phosphorus, and total potassium, and significantly positively correlated with bulk density (Figure 10).

## 4. Discussion

Fungal groups were less sensitive to salt stress than bacteria [18]. This study analyzed the diversity and richness of fungal colonies in different comparison groups. The results showed that there was no significant change in the diversity and richness of fungal colonies in different substrates under salt stress compared with their respective non-salt controls.

Ascomycota fungi are essential drivers in carbon and nitrogen cycling in arid ecosystems. These fungi play roles in soil stability, plant biomass decomposition, and endophytic interactions with plants [19]. According to the community composition analysis, the responses of Ascomycota to salt stress in different substrates were different. Specifically, this study found that the relative abundance of Ascomycota increased in TY1, TY2, and TY4, and decreased in TY3 compared with their respective non-salt controls. A previous study found that the relative abundance of Ascomycota was reduced under salt stress [20]. The differences in the responses of Ascomycota to salt stress in different substrates may be caused by multiple factors, and their ecological significance is also worth exploring. This study speculated that saline conditions may be more suitable for the growth of some Ascomycota in soil environments similar to S1, S2, and S4. The response patterns of Ascomycota to salt stress can provide a reference for the remediation of saline soils.

The genus *Penicillium* often plays an important role in the rhizosphere soil. Some *Penicillium* species produce solubilized phosphorus, siderophore, and phytohormones such as indole acetic acid and gibberellic acid, which are important for plant health [21,22,23,24]. The results of this study showed that the relative abundance of *Penicillium* in TY1 decreased slightly; however, those in TY2, TY3, and TY4 increased to varying degrees compared with their respective non-salt controls, and TY4 exhibited the highest level of increase. A previous study reported that *Penicillium* plays a role in increasing salt stress tolerance in various plant species [25], which provided ideas for regulating crop salt tolerance through microorganisms. For example, *Penicillium* can be inoculated in saline soil to observe its regulatory effect on crop growth and yield, thus promoting the transformation of theory into practice. The correlation analysis results showed that *Penicillium* was significantly positively correlated with total porosity, total nitrogen, total phosphorus, and total potassium, and significantly negatively correlated with bulk density. This study speculated that increasing total porosity might promote the ecological functions of *Penicillium*. High porosity soils facilitate oxygen diffusion, allowing for aerobic fungi such as *Penicillium* to grow and metabolize better. However, not all fungal communities had such a relationship with soil porosity. The results of this study showed that *Acrostalagmus* was significantly negatively correlated with total porosity under non-salt conditions. Members of *Acrostalagmus* are known for their ability to produce a variety of secondary metabolites [26]. This negative correlation between *Acrostalagmus* and total porosity suggest that *Acrostalagmus* may be more adapted to low-oxygen environments.

Lefse multi-level species discrimination analysis showed that *Trichoderma* and Helotiales were enriched in TY3. According to reports, *Trichoderma* can be used to control soil-borne diseases in various plants [27]. Helotiales are confirmed as a key lineage mediating nutrient acquisition by plants [28]. Many studies reported that inoculating plants with Helotiales strains can enhance plant growth [28,29,30]. Meanwhile, this study found Dothideomycetes were enriched in TY1. According to the report, they help plant development by protecting the host from stress conditions and supplying nutrients [31,32]. Additionally, an important fungal community, Chytridiomycota, was enriched in T4. This is a representative community in T4 due to its large LDA score. The Chytridiomycota phylum contributes to nutrient cycling [33] and plays an important role in soil ecosystems. Another fungal community enriched in T4 was Agaricomycetes. They are widely distributed in the soil environment and participate in the decomposition and transformation of soil organic matter [34,35,36,37]. In addition, this study found that Ascomycota, *Moesziomyces*, and Plectosphaerellaceae were the fungal colonies with the highest LDA scores in T3, T2, and T1, respectively. These Lefse multi-level species difference discrimination results can identify biomarkers in different treatments and reveal the core communities in response to substrate changes or salt stress.

## 5. Conclusions

This study set up four substrates with different bulk density, total porosity, and nutrient levels by adjusting the sand, peat moss, and laterite ratio. The results identified biomarkers in different treatments and preliminarily revealed the differential responses of rice rhizosphere fungal colonies to salt stress in different substrates and the relationship between these colonies and different physicochemical parameters. The results of this study contribute to a deeper understanding of the ecological functions of fungi.

## Figures and Tables

**Table 1:** Different treatments.

Substances	Treatments	Symbols
S1	Fresh water	T1
S2	Fresh water	T2
S3	Fresh water	T3
S4	Fresh water	T4
S1	Salt stress	TY1
S2	Salt stress	TY2
S3	Salt stress	TY3
S4	Salt stress	TY4

**Table 2:** Primer design.

Sequencing Region	Primer Name	Primer Sequences (5′-3′)
ITS1F_ITS2R	ITS1F	CTTGGTCATTTAGAGGAAGTAA
	ITS2R	GCTGCGTTCTTCATCGATGC

## Data Availability

The data presented in this study are available on request from the corresponding author.
